# Tuberculosis incidence among infected contacts detected through contact tracing of smear-positive patients

**DOI:** 10.1371/journal.pone.0215322

**Published:** 2019-04-15

**Authors:** Mario Martin-Sanchez, Silvia Brugueras, Anna de Andrés, Pere Simon, Pilar Gorrindo, Miriam Ros, Eva Masdeu, Joan-Pau Millet, Joan A. Caylà, Àngels Orcau, the Contact Tracing Group of the Tuberculosis Investigation Unit of Barcelona

**Affiliations:** 1 Epidemiology Service, Agència de Salut Pública de Barcelona (ASPB), Barcelona, Spain; 2 Preventive Medicine and Public Health Training Unit Parc de Salut Mar–Pompeu Fabra University—Agència de Salut Pública de Barcelona (PSMar-UPF-ASPB), Barcelona, Spain; 3 The Biomedical Research Center Network of Epidemiology and Public Health (CIBERESP), Madrid, Spain; 4 Department of Paediatrics, Obstetrics and Gynaecology, and Preventive Medicine, Universitat Autònoma de Barcelona, Barcelona, Spain; 5 Foundation of the Tuberculosis Research Unit of Barcelona (fuiTB), Barcelona, Spain; Chinese Academy of Medical Sciences and Peking Union Medical College, CHINA

## Abstract

**Background:**

The contacts of people with pulmonary tuberculosis (PTB) have a high risk of becoming infected and developing tuberculosis (TB). Our aim was to determine the incidence of TB and its risk factors in a cohort of contacts with latent TB infection (LTBI) detected through contact tracing of smear-positive PTB cases.

**Methods and findings:**

We performed a population-based retrospective cohort study including contacts that had LTBI, and were contacts of people with PTB who started treatment between 2008 and 2014. We followed up contacts until they developed TB or until the end date for follow-up (31st December 2016). We used Kaplan-Meier curves to compute incidence at 2 and 5 years, and Cox regression to compute hazard ratios (HR) and their 95% confidence intervals (CI). We analyzed 3097 close contacts of 565 PTB cases. After exclusion of 81 co-prevalent TB cases, 953 contacts had LTBI, of which 14 developed TB. Their risk of developing TB after two and five years was 0.7% (CI: 0.3–1.6) and 1.8% (CI: 1.1–3.1) respectively. Contacts who had not been referred for LTBI treatment had a 1.0% (CI: 0.2–4.0) risk at 5 years. Risk of developing TB at 5 years was 1.2% (CI: 0.5–3.0) among people who completed treatment, and 11.1% (CI: 5.1–23.3) for those who did not. Risk factors for TB were not completing LTBI treatment (HR 9.4, CI: 2.9–30.8) and being female (HR 3.5, CI: 1.1-11-3).

**Conclusions:**

LTBI treatment plays a fundamental role in decreasing the risk of developing TB. It is necessary to achieve a maximum contact tracing coverage and the highest possible compliance with LTBI treatment.

## Introduction

The contacts of people with tuberculosis (TB) have higher risk of having a latent tuberculosis infection (LTBI), and of developing TB than the general population. This is particularly true among the contacts of people with sputum smear-positive pulmonary TB (SS+ PTB). [1]

People with LTBI have a 5–10% lifetime risk of developing TB if they do not receive LTBI treatment. The risk is higher in the first two years after infection and decreases gradually thereafter.[2] Other factors can also increase the risk of developing TB, particularly diseases or conditions that reduce or impair the function of the immune system.[3]

Recent studies performed in countries with a low incidence of the disease have shown that the risk of developing TB within 5 years after contracting LTBI could be as high as 9.5%[4] or even 15%[5]. Several studies performed in different settings have obtained different risks at the population level.[1,4–8]

To decrease infected individuals’ risk of developing TB, LTBI treatment prescription is considered.[9] In our setting, this treatment usually consists of one of the following: (i) six- to nine-month treatment with Isoniazid, or (ii) a 3-month treatment with a combination of Rifampin and Isoniazid[10,11]. However, TB prevention among contacts with LTBI depends on the acceptance and completion of a long-term treatment. While clinical trials have shown that LTBI treatment has around 90% efficacy[12], there is broad variation in the observed rates of treatment completion in our control program. In Barcelona city, completion rates reach 70–80%[13], but are much lower in other areas [14], which reduces treatment effectiveness and increases the risk of developing TB[15].

To improve LTBI treatment indication, and to more accurately identify population groups at risk of developing TB, it is necessary to better understand the characteristics of the TB cases’ contacts[1]. The aim of this study was to determine the incidence of TB and its risk factors in a cohort of patients with LTBI who were detected by tracing the contacts of SS+ PTB cases in the city of Barcelona. This was achieved thanks to an established TB surveillance and control system in a low-medium TB incidence European city[16].

## Methods

### Design

We performed a population-based retrospective cohort study of infected people who were contacts (intensity of contact at least one hour per day) of SS+ PTB cases who had initiated treatment between 2008 and 2014. Contacts were detected through the Barcelona TB Control Program (PCTB). The analysis was carried out retrospectively and involved data collected on a routine basis within the National Tuberculosis Plan approved by the Spanish Ministry of Health. Therefore, informed consent was not required.

### Study population

We selected close and frequent contacts of SS+ PTB index cases obtained through the PCTB contact registry. Potential contacts were excluded if their corresponding index case was not living in Barcelona when the contacts were traced.

Data were collected for socio-demographic (sex, country of origin and date of birth) and clinical and epidemiological variables (final result of contact tracing, prescription and completion of LTBI treatment) registered by the PCTB. Contacts were defined as having completed treatment if they followed the prescribed treatment with good adherence as determined by the corresponding physician until (i) the treatment was completed, or (ii) the end date for follow-up was reached. Contacts were classified as non-completers if they refused the prescribed treatment, dropped out voluntarily, or discontinued treatment for toxicity-related or unspecified reasons. For contacts in which some results were missing, some socio-demographic data were obtained from the city census. The final follow-up date for contacts who had left the city or had died were also obtained from the city census. Other clinical variables of interest were obtained by retrospectively reviewing hospital and primary care records using standardized abstraction forms used by the PTCB. These included: (i) smoking habit, (ii) alcohol abuse (alcohol consumption over 280 gr per week in men and over 140 gr in women), (iii) diabetes mellitus (DM), and (iv) infection with human immunodeficiency virus (HIV).

### Field methods

All newly diagnosed TB cases are notified to the PCTB for evaluation. Contact tracing is prioritized according to the following factors: (i) level of infectiousness of the index case; (ii) intensity of exposure; (iii) characteristics of the place of exposure; (iv) the risk of developing TB. Contact tracing is prioritized for SS+ PTB cases.

Contacts are classified as follows: (i) close contacts (in contact every day for ≥6 hours), (ii) frequent contacts (in contact for at least 1 hour per day), (iii) non-daily contacts (in contact for at least 6 hours a week), and (iv) sporadic contacts. For SS+ PTB cases, contact tracing was stipulated for any contacts that occurred in the 3 months prior to the onset of symptoms in the index case. The intensity of contact is matched with the risk of developing TB in order to create high-, medium- and low- priority groups[17].

High-priority contact tracing is conducted as soon as possible after diagnosis of the index case. Contact tracing at this stage involves collecting some basic socio-demographic data (name, surname, country of origin, sex, date of birth), performing a brief anamnesis, and analyzing the presence of LTBI by performing a tuberculin skin test (TST) injection or by using interferon gamma detection techniques (IGRA, QuantiFERON-TB[18]), depending on the risk of infection or on the presence of immunosuppression. For contacts of TB patients, LTBI treatment is considered if the TST results in a 5-mm induration (or higher), or if the Quantiferon-TB result is 0.35IU/ml or higher[19].

For all contacts traced, the test is repeated at weeks 8 and 12 -exceeding the window period- if the most recent exposure to the index case occurred <8 weeks previously. This allows us to detect any conversions in LTBI tests; conversions were defined as a change from a negative to a positive value in the TST or IGRA, according to the thresholds defined above[19].

In all cases with an infection, the presence of the disease was assessed by evaluation of the individual clinical history and a chest X-ray. The contacts diagnosed with active TB disease at this stage were classified as co-prevalent TB cases. Once absence of disease is confirmed, the clinician recommends either LTBI treatment or clinical follow-up. LTBI treatment is offered to all infected contacts of TB cases, especially those aged <65 years. The corresponding clinician assesses each case individually, according to the epidemiological and clinical characteristics of the person, in order to decide on the best treatment regimen, if any.

### Follow-up of the study population

Contacts were followed up from the day the index case started treatment until they developed TB, or until December 31^st^, 2016. Contacts who were lost to follow-up or who had left the country were censored at the last date registered in the clinical history; contacts who had died were censored at the date of death as recorded in the city census.

The dependent variable was time to TB diagnosis, which was obtained after subtracting the final date of follow-up from the date at which the index case started treatment. It was obtained by cross-checking the PCTB contacts register against the PCTB register of TB cases that has started treatment between 2008 and 2016. To confirm that the data corresponded to the same person, we created an individual coded variable including name, surname and date of birth in both databases using a common algorithm. Afterwards, all matching cases were verified by the PCTB staff. In addition, we searched for all contacts in electronic clinical records in order to detect any case that could have developed TB outside Barcelona but within Catalonia.

### Statistical analysis

We computed TB incidence at two and five years using Kaplan-Meier curves, globally and stratified by sex, age (which was re-coded into two groups, <40 years and ≥40 years, in order to eliminate empty categories), DM, smoking habit, HIV infection, risky alcohol consumption, country of origin (born in Spain, or born in a another country), conversion of TST/IGRA, and prescription and completion of LTBI treatment (treatment completers, non-completers, no prescription, or unknown). We used the Log-Rank and Wilcoxon tests for hypothesis contrasting, and set statistical significance at p<0.05.

We analysed missing values for the prescription and completion of LTBI treatment, comparing the known and missing values for this variable. For contacts who had been prescribed LTBI treatment, we compared those who completed the treatment to those who did not using the χ² and Fisher exact tests, with statistical significance set at p<0.05. We used logistic regressions to calculate the odds ratio (OR) and 95% confidence interval (CI), and fit multivariate models for all variables, excluding (stepwise backwards) those with a larger p-value than 0.2.

We computed the density of incidence (and 95%CI) in 100,000 persons-year of follow-up (PY), both globally and stratified by prescription and completion of LTBI treatment. To identify risk factors for developing TB, we used a multivariate Cox regression analysis to compute adjusted hazard ratios (aHR) and the corresponding confidence intervals. We fit a model with all variables, and then excluded (stepwise backward) those with a p-value of >0.2. Age and sex were maintained in the model, as stipulated by the theoretical framework. For the variable “prescription and completion of LTBI treatment”, all missing values were included in the model as a category. The proportionality of the risk was tested over time using Schoenfeld residuals[20].

Starting with the multivariate model, we used variance-normalizing and stabilizing- transformations to compute the etiologic fraction for developing TB and its confidence intervals for non-compliance with LTBI treatment[21].

All analysis were performed using STATA (13.0 Version; Stata Corp, College Station, TX).

### Ethical considerations

This study was approved by the Clinical Research Ethics Committee of Parc de Salut Mar (CEIC-IMAS) in Barcelona (Project number 2017/7443/I).

To guarantee confidentiality of the data and records, we adhered to the regulations established by Spanish Organic Law 15/1999 on the Protection of Personal Data, and to the Ethical Principles for Human Research defined by the 1964 Declaration of Helsinki, revised and updated by the World Medical Organization (Edinburgh 2000).

## Results

### Characteristics of the study population

**Fig 1:**
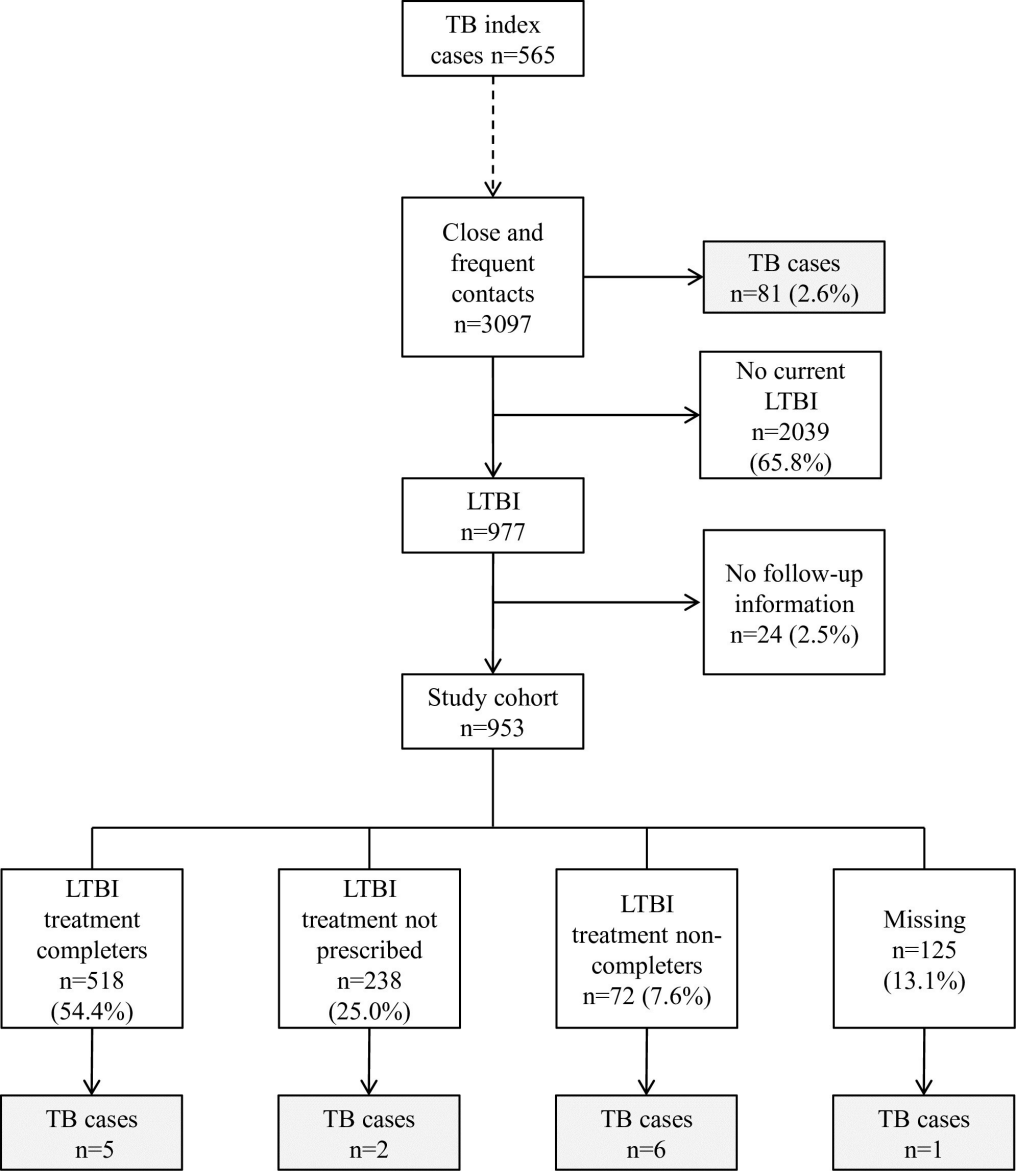
Results obtained from tracing and follow-up of close and frequent contacts of sputum smear-positive pulmonary tuberculosis cases. **Barcelona, 2008–2016. (**TB: tuberculosis, LTBI: latent tuberculosis infection).

A total of 2637 TB cases were detected in the study period, of which 697 had SS+ PTB. 565 cases (81.1%) were people who were living in Barcelona and had at least one close or frequent contact captured by the contact tracing and for whom follow-up data were available. For these 565 cases, we captured 3,097 contacts, of whom 81 (2.6%) were found to have co-prevalent TB, and 977 (31.6%) had LTBI; of the latter group, 92 had a TST or IGRA conversion (Fig 1).

Among the contacts with either co-prevalent TB or LTBI, the proportion of contacts that had co-prevalent TB in each age group was as follows: 60.7% in <5 years, 20.9% in 5–14 years; 6.9% in 15–39 years and 2.2% in ≥40 years.

After excluding the co-prevalent cases, follow-up information was obtained from 953 (97.5%) contacts with LTBI who conformed our study cohort; of whom 57.2% were men, 56.5% had been born in a foreign country, and 58.8% were <40 years old. Most were close contacts (792, (83.1%)) or household contacts (735 (77.1%)).

The prevalence of other clinical variables of interest was: smoking habit, 25.6%; diabetes, 5.6%; alcohol abuse, 5.1%, and HIV infection, 0.3%. In terms of LTBI treatment: this treatment was not prescribed in 238 (25.0%) cases, treatment information was unavailable in 125 (13.1%) cases, treatment was completed in 518 (54.4%) cases, and 72 (7.6%) of the cases did not comply with treatment (Table 1).

**Table 1:** Characteristics of the 953 infected contacts (close and frequent contacts) detected through contact tracing of sputum smear-positive pulmonary tuberculosis cases. Barcelona 2008–2016.

		n (%)
Age	<5 years	11 (1.2)
	5–14 years	66 (6.9)
	15–39 years	483 (50.7)
	≥40 years	392 (41.1)
	Unknown	1 (0.1)
Sex	Men	545 (57.2)
	Women	408 (42.8)
Country of origin	Spain	399 (41.9)
	Foreign-born	539 (56.5)
	Unknown	15 (1.6)
Type of contact	Close	792 (83.1)
	Frequent	161 (16.9)
Setting	Home	735 (77.1)
	Community	218 (22.9)
TST/IGRA Conversion	Yes	92 (9.7)
	No	861 (90.3)
Smoking habit	Yes	244 (25.6)
	No	481 (50.5)
	Missing	228 (23.9)
DM	Yes	53 (5.6)
	No	732 (76.8)
	Missing	168 (17.6)
HIV infection	Yes	3 (0.3)
	No	788 (82.7)
	Missing	162 (17.0)
Alcohol abuse	Yes	49 (5.1)
	No	668 (70.1)
	Missing	236 (24.8)
Prescription and completion of LTBI treatment	Completers	518 (54.4)
	No prescription	238 (25.0)
	Non-completers	72 (7.6)
	Missing	125 (13.1)

TST/IGRA conversion: conversion in tuberculin screen test or IGRA seroconversion; DM: diabetes mellitus; LTBI: latent tuberculosis infection

The global rate of treatment completion among those commencing treatment was 87.8%, with the highest rates observed in children aged 5–14 years (98.4%), followed by children aged <5 years (90.9%), contacts aged ≥40 years (89.9%), and contacts aged 15–39 years old (84.2%). Non-completion of LTBI treatment was due to treatment refusal by 16 (22.2%) contacts, voluntary drop-out in 25 (34.7%) contacts, toxicity in 9 (12.5%) contacts, and non-specified reasons in 22 (30.6%) contacts.

The median age of people who completed LTBI treatment (treatment completers) was 34 years, and 34.6% of them were ≥40 years old. The median age of non-completers was 32.5 years, and 27.8% of them were ≥40 years old. The median age of people who were not given a prescription for treatment was 45 years, and 63.0% of them were older than ≥40 years of age.

Most contacts for whom no data were available regarding prescription and completion of LTBI treatment were foreign-born, non-household contacts and non-converters in the LTBI tests (Table 2).

**Table 2:** Factors associated with missing data regarding prescription and completion of latent tuberculosis infection treatment. 953 infected contacts (close and frequent contacts) detected through contact tracing of sputum smear-positive pulmonary tuberculosis cases. Barcelona 2008–2016.

		Prescription and completion of LTBI treatment				
		Missing	Non-missing	p-value**	OR	aOR
		[n (%*)]	[n (%*)]			
Age	<40 years	82 (14.6)	478 (85.4)	0.10	1	
	≥40 years	43 (11.0)	349 (89.0)		0.7 (0.5–1.1)	
Sex	Man	77 (14.1)	468 (85.9)	0.29	1	
	Woman	48 (11.8)	360 (88.2)		0.8 (0.6–1.2)	
Country of origin	Spain	31 (7.8)	368 (92.2)	<0.01	1	1
	Foreign-born	93 (17.3)	446 (82.7)		2.5 (1.6–3.8)	2.3 (1.3–3.8)
Type of contact	Close	104 (13.1)	688 (86.9)	0.98	1	
	Frequent	21 (13.0)	140 (87.0)		1.0 (0.6–1.6)	
Setting	Home	89 (12.1)	646 (87.9)	0.09	1	1
	Community	36 (16.5)	182 (83.5)		1.4 (0.9–2.2)	2.4 (1.4–4.1)
TST/IGRA conversion	Yes	3 (3.3)	89 (96.7)	<0.01	1	1
	No	122 (14.2)	739 (85.8)		4.9 (1.5.15.7)	5.4 (1.3–22.9)
Smoking habit	No	50 (10.4)	431 (89.6)	0.21	1	1
	Yes	33 (13.5)	211 (86.5)		1.3 (0.8–2.1)	1.4 (0.9–2.4)
DM	No	85 (11.6)	647 (88.4)	0.73	1	
	Yes	7 (13.2)	46 (86.8)		1.2 (0.5–2.6)	
HIV infection	No	95 (12.1)	693 (87.9)	0.52		
	Yes	0 (0.0)	3 (100)			
Alcohol abuse	No	78 (11.7)	590 (88.3)	0.74	1	
	Yes	5 (10.2)	44 (89.8)		0.9 (0.3–2.2)	

LTBI: latent tuberculosis infection; OR: odds ratio; aOR: odds ratio adjusted by country of origin, setting of contact, TST/IGRA conversion and smoking habit; TST/IGRA conversion: conversion in tuberculin screen test or IGRA seroconversion; DM: diabetes mellitus.

*Percentage over the total without missing values;

**p-value for the univariate analyses.

Among contacts who were prescribed LTBI treatment, those who did not complete it were more often foreign-born than those who did (Table 3).

**Table 3:** Factors associated with non-completion of latent tuberculosis infection treatment. 590 contacts (close and frequent contacts) to whom treatment had been prescribed; contacts were detected through contact tracing of sputum smear-positive pulmonary tuberculosis cases. Barcelona 2008–2016.

		LTBI treatment				
		Non-completion	Completion	p-value**	OR	aOR
		n = 72, n (%*)	n = 518, n (%*)			
Age	<40 years	52 (13.3)	338 (86.7)	0.25	1	
	≥40 years	20 (10.1)	179 (89.9)		0.7 (0.4–1.3)	
Sex	Man	40 (12.1)	291 (87.9)	0.92	1	
	Woman	32 (12.4)	227 (87.6)		1.0 (0.6–1.7)	
Country of origin	Spain	20 (8.3)	222 (91.7)	0.02	1	1
	Foreign-born	50 (14.7)	290 (85.3)		1.9 (1.1–3.3)	2.0 (1.0–3.7)
Type of contact	Close	58 (11.4)	452 (88.6)	0.12	1	
	Frequent	14 (17.5)	66 (82.5)		1.6 (0.9–3.1)	
Setting	Home	55 (10.9)	449 (89.1)	0.02	1	1
	Community	17 (19.8)	69 (80.2)		2.0 (1.1–3.7)	1.8 (0.8–3.9)
TST/IGRA Conversion	Yes	4 (5.9)	64 (94.1)	0.09	1	
	No	68 (13.0)	454 (87.0)		2.4 (0.8–6.8)	
Smoking habit	No	34 (10.1)	303 (89.9)	0.59	1	
	Yes	16 (11.8)	120 (88.2)		1.2 (0.6–2.2)	
DM	No	48 (10.1)	428 (89.9)	0.25	1	
	Yes	5 (16.7)	25 (83.3)		1.8 (0.6–4.9)	
HIV infection	No	53 (10.4)	456 (89.6)	0.07	1	
	Yes	1 (50.0)	1 (50.0)		8.6 (0.5–139.6)	
Alcohol abuse	No	48 (11.0)	389 (89.0)	0.53	1	
	Yes	2 (7.1)	26 (92.9)		0.6 (0.1–2.7)	

LTBI: latent tuberculosis infection; OR: odds ratio; aOR: odds ratio adjusted by country of origin and setting of contact; TST/IGRA conversion: conversion in tuberculin screen test or IGRA seroconversion; DM: diabetes mellitus.

*Percentage over the total without missing values;

**p-value for the univariate analyses;.

### Incidence of tuberculosis

**Fig 2:**
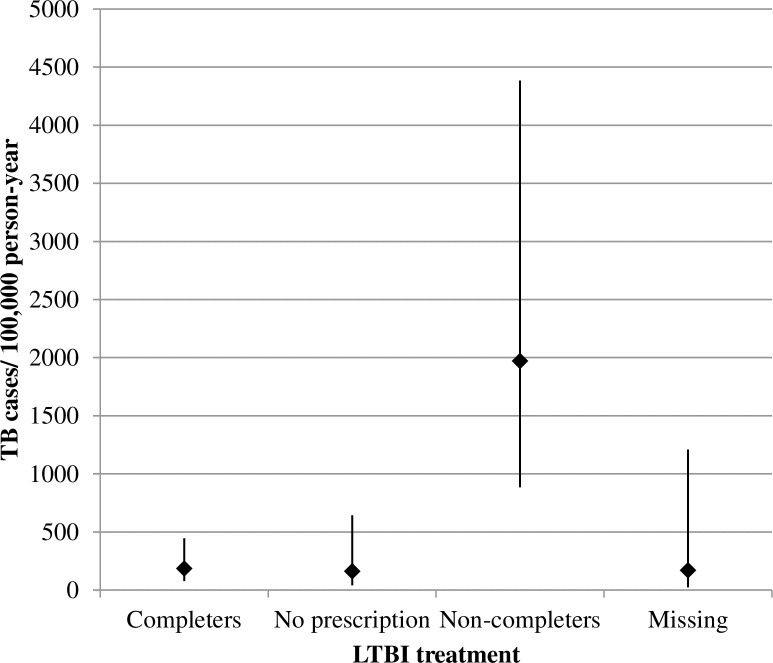
Incidence of tuberculosis among 953 infected contacts (close and frequent contacts) detected through contact tracing of sputum smear-positive pulmonary tuberculosis cases. **Results stratified by prescription and completion of latent tuberculosis infection treatment. Barcelona 2008–2016.** Absolute value and 95% confidence intervals; TB: tuberculosis; LTBI: latent tuberculosis infection.

We detected 14 cases of TB with a median contact follow-up time of 5.3 years (interquartile range = 2.9–7.7). The median age of the cases was 31 years (range, 19–72 years). The incidence density was 290.1 TB cases/100.000 PY (CI: 171.8–489.9) and there were no significant differences between groups, except in terms of “prescription and completion of LTBI treatment” (p<0.01): 185.7 cases/1000,000 PY (CI: 77.3–446.1) in treatment completers, 1969.9 (CI: 885.0–4,384.9) in treatment non-completers, 161.0 (CI: 40.3–643.9) in contacts who had not been prescribed treatment, 170.5 (CI: 24.0–1210.4) (Fig 2) in contacts for whom no information was available regarding treatment prescription and completion.

At the univariate level, TB risk was significantly higher in individuals who had not completed LTBI treatment (HR 9.8, CI: 3.0–32.4, compared to treatment completers). Women (HR 3.0; CI: 0.9–9.7) and foreign-born individuals (HR 3.3; CI: 0.9–11.8) had marginally significantly greater risk of TB. Similar results were obtained in the multivariate analysis (aHR 9.4, CI:2.9–30.8 in treatment non-completers, and aHR 3.5 CI:1.1–11.3 in women; Table 4).

**Table 4:** Risk factors for tuberculosis among 953 infected contacts (close and frequent contacts) detected through contact tracing of sputum smear-positive pulmonary tuberculosis cases. Barcelona 2008–2016.

		Incident TB cases (n)	% TB risk at 5 years (95% CI)	HR (CI95%)	aHR (CI95%)
Age	<40 years	10	2.4 (1.3–4.4)	1	1
	≥40 years	4	1.1 (0.4–3.0)	1.0 (0.9–1.0)	0.8 (0.5–1.5)
Sex	Man	4	1.1 (0.4–2.9)	1	1
	Woman	10	2.8 (1.5–5.2)	3.0 (0.9–9.7)	3.5 (1.1–11.3)
Country of origin	Spain	3	0.8 (0.3–2.4)	1	
	Foreign-born	11	3.0 (1.6–5.3)	3.3 (0.9–11.8)	
TST/IGRA Conversion	No	12	1.8 (1.0–3.1)	1	
	Yes	2	2.6 (0.7–10.0)	1.5 (0.3–6.8)	
Smoking habit	No	5	2.1 (1.1–4.1)	1	
	Yes	9	2.3 (0.9–5.3)	1.1 (0.4–3.4)	
DM	No	12	1.9 (1.1–3.3)	1	
	Yes	2	4.1 (1.0–15.6)	2.2 (0.5–10.0)	
HIV infection	No	14			
	Yes	0			
Alcohol abuse	No	14			
	Yes	0			
Prescription and completion of LTBI treatment	Completers	5	1.2 (0.5–2.9)	1	1
	No prescription	2	1.0 (0.3–4.0)	0.9 (0.2–4.5)	1.2 (0.2–6.3)
	Non-completers	6	11.2 (5.1–23.3)	9.8 (3.0–32.2)	9.4 (2.9–30.8)
	Missing	1	1.0 (0.1–6.8)	1.0 (0.1–8.2)	0.9 (0.1–7.7)

HR: hazard ratio; aHR: hazard ratio adjusted by age, sex and prescription and completion of LTBI treatment and stratified by country of origin as proportional hazards assumption was not complied (N = 952); TST/IGRA conversion: tuberculin screen test conversion or IGRA seroconversion; DM: diabetes mellitus; LTBI: latent tuberculosis infection.

### Cumulative incidence at 2 and 5 years

**Fig 3:**
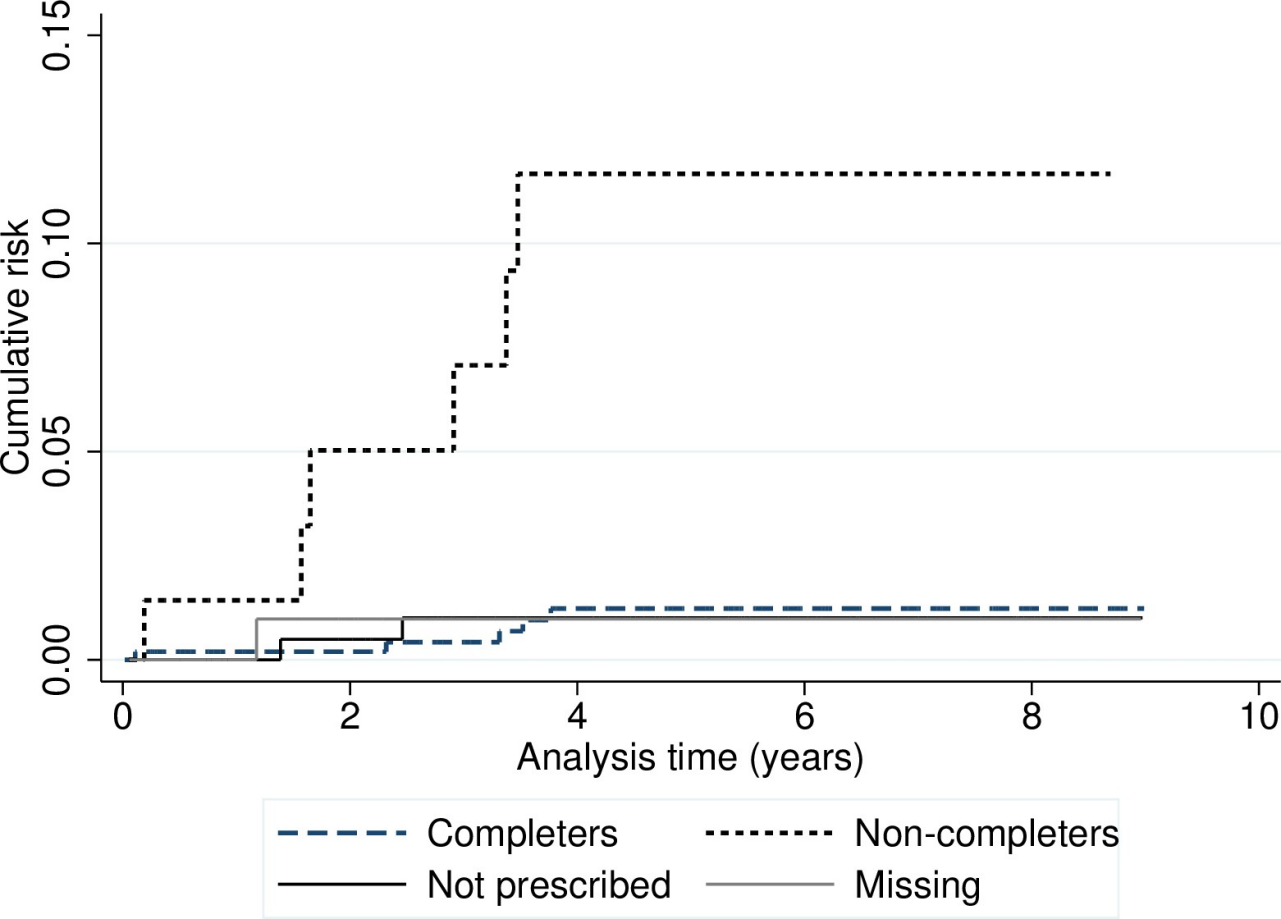
Cumulative risk of tuberculosis in 953 infected contacts (close and frequent contacts) detected through contact tracing of sputum smear-positive pulmonary tuberculosis cases stratified by prescription and completion of latent tuberculosis infection treatment. Barcelona 2008–2016.

For all contacts, the overall probability of developing TB within two years was 0.7% (CI: 0.3–1.6). There were no statistically significant differences in risk between categories, except in relation to “prescription and completion of LTBI treatment”: 0.2% (CI: 0.1–1.4%) in treatment completers; 0.5% (CI: 0.1–3.4%) in contacts who had not been prescribed treatment; 5.0% (CI: 1.6–14.7%) in non-completers; and 1.0% (CI: 0.1–6.8%) in contacts for whom no information was available regarding the prescription and completion of LTBI treatment (Fig 3).

For all contacts, the overall probability of developing TB within five years was 1.8% (CI: 1.1–3.1). There were no statistically significant differences in risk between categories, except for the prescription and completion of LTBI treatment: 1.2% (CI: 0.5–2.9%) in contacts who completed LTBI treatment; 1.0% (CI: 0.2–4.0%) in contacts who had not been prescribed treatment; 11.2% (CI: 5.1–23.3%) in non-completers; and 1.0% (CI: 0.1–6.8%) in contacts for whom no information was available regarding the prescription and completion of LTBI treatment (Table 4 and Fig 3).

In terms of TB development, the etiologic fraction of contacts who had not completed LTBI treatment was 89.6% (CI: 65.7–96.9), i.e. 5 of the 6 TB cases who had not completed LTBI treatment would have been avoided if they had completed their treatment.

## Discussion

This study shows that contacts with LTBI who were detected by tracing contacts of SS+ PTB patients have a high incidence of TB during follow-up, which makes them a high-risk group. In these cases, the incidence reached 290.1 cases per 100,000 PY, which is 17 times higher than the global incidence in Barcelona in the year 2016 (16.2 cases per 100,000 PY[13]).

Of all the contacts mentioned above, the highest-risk group was composed of contacts who had been prescribed LTBI treatment but did not complete it. Contacts in this group had 10-fold greater risk of developing TB than those who completed the treatment. In the contacts who did not complete treatment, the risk of developing tuberculosis was 5% at 2 years, and 11.2% at 5 years. In contrast, the risk was close to 1% among contacts who had not been prescribed treatment and in those who had completed LTBI treatment. These values are somewhat higher than estimated classically [2], and some of the results are comparable to those obtained in recent studies performed in low- and medium-incidence countries [4,5,22]. For instance, in our study we observed an incidence of 1969.9 cases/100,000 PY in contacts who had not completed treatment, while a study performed in Canada found an incidence of 1494 cases/100,000 PY[22] in the same group.

Our results also confirm that prescription and completion of LTBI treatment are effective in reducing the TB risk, since nearly 90% of the cases in which non-completer contacts developed TB would have been avoided if they had actually completed the treatment prescribed. In addition to the cases that would have been prevented in the short term, secondary cases of these contacts would also have been prevented, as contact tracing makes it possible to break the chain of infection.[1] Remarkably, there were no differences in incidence between contacts who had not been prescribed LTBI treatment and those who had been prescribed the treatment and completed it. Even though all close contacts with LTBI are generally advised to follow treatment, various factors may render treatment inappropriate for medical reasons, such as: (i) suspected previous infection, which lowers the risk of reactivation [23]; (ii) co-morbidities that make the patient more vulnerable to the adverse effects of treatment[12]; and (iii) suspected poor adherence to treatment where prescribed. In light of these results, the contacts to whom treatment was not prescribed were generally older than those who were prescribed treatment, and probably had previous TB infections with a much lower risk of disease reactivation.

Another result of this study was the higher TB risk observed in women, even though this result was marginally significant. Some TB cases in men may have occurred after the end of the study period, since they had a shorter total follow-up than women; in fact, a single additional case of TB in men would have made the gender difference statistically non-significant. Men and women may be expected to have a similar risk of TB after adjusting for clinical and socio-demographical variables. Nevertheless, while at least one study reported higher risk in women [24], others have reported higher risk in men [25,26]. More work is necessary to definitively determine whether there is a real gender difference in risk and its determinants.

A detailed analysis of the prescription and completion of LTBI treatment showed that in most cases contacts with missing values were foreign-born, community contacts of the index case, or non-converters in LTBI tests. These contacts had a similar profile to that of contacts who did not complete LTBI treatment, which could underestimate the incidence of TB in the total number of contacts.

Among foreign-born contacts, the lower treatment completion and higher percentage of individuals lost to follow-up suggest that this is an especially vulnerable group, for which specific strategies should be developed to improve follow-up and increase adherence. Programs that encourage linguistically- and culturally- sensitive care, free access to treatment, shorter treatments, and individualized care have proven useful in increasing compliance in this population [14,27,28].

The study has several limitations. First, we had limited access to information related to several factors that could have influenced the risk of TB such as the presence of immunosuppression due to diseases and other treatments [1]. Also, data were missing for some risk factors such as smoking habit, alcohol abuse, HIV infection and diabetes, as these are not systematically registered in contact follow-up. This issue, along with the small number of incident cases, may explain that none of the risk factors mentioned above increased TB risk in our cohort. Specifically, none of the 14 incident TB cases had HIV infection or a history of alcohol abuse, and while we found that DM was associated with a higher risk of TB, this association was not statistically significant.

Second, the sample size and the lack of incident TB cases among contacts under 15 years of age only allowed us to create two age groups for the regression models. Even when risk is higher in contacts aged <15 years, this was not shown in follow-up since they had already appeared at the beginning of the contact tracing. Due to their vulnerability, TB cases in this age group probably appear even before the diagnosis of the index case. In our study population, a 60.7% of the contacts with either active TB or LTBI at the baseline contact tracing were co-prevalent TB cases in <5 years old, as compared to the 2.2% in ≥40 years old.

Third, only infected contacts were followed up in this study. Even though TST and IGRA are the recommended screening tests [19], they do not have optimal sensitivity [29] and some infected contacts might be misclassified as non-infected and therefore excluded, when in fact they are infected and have high TB risk.

Finally, the only contacts included were those of cases in which contact tracing was successful. This means that we do not have information on the characteristics and the TB risk of contacts that could not be traced or identified by the PCTB. Nevertheless, this proportion was low in SS+PTB cases, and would likely have little influence on the results.

This study also has several strong points. First, it is one of the few population-based studies to assess the incidence of TB at a community level. Second, it includes the complete records of cases and contacts registered by the Barcelona PCTB, which is an organized, expert system that ensures all new cases are registered, thus minimizing case loss and non-detection[16]. Third, we had access to shared clinical registers covering all of Catalonia, which allowed us to double-check the cases. Fourth, we had a long follow-up period for contacts, which in most cases included the periods in which TB risk was higher.

This study tackles an especially relevant topic, as countries with low TB incidence have proposed to achieve pre-elimination of this disease by 2035; according to the *End-TB* program guidelines of the World Health Organization (WHO)[30], this disease should be eliminated by 2050. Modeling studies show that treating the active disease only is insufficient to reduce TB incidence at a fast enough rate to eliminate it by 2050[31]. Unfortunately, LTBI treatment is not the standard of care in some countries. Therefore, in addition to ensuring that new TB cases are detected and treated, it is necessary to emphasize the importance of detecting and treating new LTBI [32] cases, and to develop and promote strategies that increase adherence[33]. In terms of TB research priorities for 2018, the WHO also focused on determining the incidence in at-risk populations, as this is a crucial factor for determining the potential benefits of LTBI treatment, and designing appropriate public health interventions[34].

This field requires a strong political commitment by each country to provide more resources for research, control, and surveillance programs that reinforce the role of contact tracing studies and LTBI treatment. Several strategies can be used to increase LTBI treatment compliance, such as administering directly observed treatment at the community level, introducing community health workers, or organizing clinical TB units like the one implemented in Barcelona [35–37]. Moreover, developing guidelines for new, more efficient, shorter, and better tolerated LTBI treatment regimes will also bring us closer to the WHO objectives, which now seem far away.
